# Nib-Assisted Coaxial Electrohydrodynamic Jet Printing for Nanowires Deposition

**DOI:** 10.3390/nano13091457

**Published:** 2023-04-25

**Authors:** Shiwei Shi, Zeshan Abbas, Xiangyu Zhao, Junsheng Liang, Dazhi Wang

**Affiliations:** Key Laboratory for Micro/Nano Technology and System of Liaoning Province, Dalian 116024, China; sd126ssw@mail.dlut.edu.cn (S.S.); hopenotout1214@mail.dlut.edu.cn (Z.A.); zxy4195@mail.dlut.edu.cn (X.Z.); jsliang@dlut.edu.cn (J.L.)

**Keywords:** numerical simulation, nib-assisted, coaxial electrohydrodynamic jet, identified particle

## Abstract

This paper presents the concrete design of nanowires under the precise size and morphology that play a crucial role in the practical operation of the micro/nano devices. A straightforward and operative method termed as nib-assistance coaxial electrohydrodynamic (CEHD) printing technology was proposed. It extracts the essence of a nib-assistance electric field intensity to enhance and lessen the internal fluid reflux of the CEHD jet. The experiments were performed to add microparticles into the inner liquid to indicate the liquid flow consistency within the coaxial jet. The reflux in the coaxial jet was observed for the first time in experiments. The nanowires with a minimum size of 70 nm were printed under optimum experimental conditions. The nanopatterns contained aligned nanowires structures with diameters much smaller than the inner diameter of nozzle, relying on the coaxial nib-assisted technique. The printed results revealed that the nib-assisted CEHD printing technique offers a certain level high quality for application of NEMS system.

## 1. Introduction

Different functional materials are used to fabricate particular micro/nanostructures which play a crucial role at the extensive range of practical applications in field effect transistors [1], biosensing [2,3,4] and biological tissue engineering [3,4]. Thus, the high-performance sensors [5], flexible electronic devices [6] and stretchable electronic skin [7,8] were fabricated using functional materials. Several studies were conducted on multiple ways to fabricate micro/nanowires for desired steerable arrays and patterned structures. There are many common printing techniques that mainly contain lithography [9,10], transfer printing [11,12], etching process [13,14] and inkjet printing [15]. So, lithography is preferred to nanoscale structures for higher production definition among these approaches. However, its printing capability is restricted by costly facility deficiencies and tedious process. However, the electrohydrodynamic (EHD) printing system recently became popular due to its high resolution and low cost. When a sufficient voltage is applied to the fluid, its produces shear thinning between the fluid particles and forms a high-speed jet. Electrohydrodynamic jet (E-Jet) direct writing process is a very attractive manufacturing method for preparing micro/nanostructures using atomization process for the application in many fields [16].The typical equipment (EHD) contains a high-voltage DC power source, a precision pump for liquid supply, a hollow needle for solution out-flow, a movable printing platform and an auxiliary computer with a camera. Many researchers investigated the EHD jet believer. In 1882, Rayleigh discovered that the stable liquid surface became unstable when subjected to a strong electric field and even a jet stream appeared. Later, Zeleny made a substantial contribution to this problem and studied the phenomenon of deformation and jet generation under the action of small and low early electric fields at the bottom of single capillary glass tubes [17,18]. Subsequently, analysis from Taylor’s experimental results showed that soap films and water/oil interfaces with semi-vertical angles of approximately 49.3 could be produced with calculated tension [19]. Hayati et al. proved the role of the electro-shear effect in the formation of a steady jet stream and overturned those theories which assumed that the fluid had a uniform velocity distribution at the bottom of the cone jet [20]. Tang et al. conducted an experimental study on hydroelectricity spraying in conical jet style [21]. Cloupeau et al. studied liquid with specific conductivity in single-needle electrostatic capillary spray. The diameter of the jet varies to a certain limited extent depending on the applied voltage and volume current rate. Moreover, its rate depends mainly on the geometry of the capillary [22]. Loscertales et al. presented a detailed study on compound or coaxial electrospray for biomedical device application [23].

In recent years, finite element was extensively used in the simulation of crucial process parameters in the formation of electrojet printing jets, spray, droplets and cushions jets [24]. The study added a carbon fiber to the needle to study the assistance stability during the jet development process. The work chose the FLOW3D software (FLOW3D, 2011, Flow Science, Santa Fe, NM, USA) for the modeling and simulations. Furthermore, the experimental results were well confirmed based on their assumptions [25]. The modeling and simulations for the dynamics of jets during the development of cone-jets via the software CFX 4.4 were investigated [26]. An array of micromachined ultrasonic electrosprays was numerically modeled and simulated (FLUENT CFD, 2010, ANSYS, Pennsylvania, USA) in which separate processes determine the droplet structure and the charge distribution [27]. The process of electrohydrodynamic equations through a commercial code of FLUENT v6.3 was introduced [28]. Lim et al. established a simulation model incorporating the Navier–Stokes equation and continuity equations for the gas and liquid phases. It was combined with Poisson’s equation to calculate the electrical potential [29]. Xinyun et al. simulated the impact of ESI voltage emitter tapering, flow rate, surface hydrophobicity and fluid conductivity using FLOW3D software. To achieve this goal, they combined the Taylor–Melcher leaky-dielectric model with the volume of fluid technique for tracking the fast-changing liquid-gas interface. Hayati et al. modeled the velocity field in a cone and in a jet of fixed geometry, i.e., with no free surface [30]. The values for the diameter of the jet and charge density were supposed at the onset of simulation. A finite element method was employed to solve the equations [31]. A commercial CFD code was used to simulate and study the EHD atomization process through transition of EHD equation into the simple heat transfer problem. Herrada et al. proposed a robust and computationally effective numerical scheme to simulate a stable electrohydraulic atomization process [32]. Wei et al. established the physical model of the electric field induced by charged droplets [33]. The model was combined with the charge conservation equation and the fluid flow equation to simulate the development of the cone jet. Particle tracking is widely used to study the change of fluid motion in liquid. Jihoon et al. presented the fluorescence polystyrene particles that were mixed into solution. According to the electrohydraulic jet fluorescence images obtained from the microparticle imaging velocimetry results, the flow in the Taylor cone was formed during electrohydrodynamic (EHD) spraying. It was further studied and visualized to analyze its stability in five candidate liquids [34]. Archana et al. conducted an experimental study on the Taylor cone internal and external fields by using light film fluorescence imaging and time-resolved particle imaging velocimetry. The ethanol was used as the working medium liquid [35]. Although many advances have been made, the quality and stability of the printing structure still need to be improved, and so, it is necessary to solve the stability of the printing structure and print high-performance materials to meet the needs of micro and nano sensors for high-performance nanostructures.

Lead zirconate titanate [PbZrl-xTixO3(PZT)] is a typical piezoelectric material with large remnant, high dielectric constant and electromechanical coupling coefficient [36,37]. In addition, the polarization (PZT) was widely used in the field of microelectron mechanical systems (MEMS) due to its excellent piezoelectric properties [38,39,40]. Equally, ceramic materials, bulk and thin film PZT structures are considered extremely fragile [36]. However, unlike bulk, thin films PZT structures, PZT nanofibers prepared by electrospinning exhibit high piezoelectric voltage constant, high mechanical strength and flexibility [41]. Therefore, the PZT nanofiber was very promising for manufacture of nano/micro devices in the future.

In this study, we introduce a novel nib-assisted coaxial EHD printing technique to reduce internal liquid reflux and improve the stability of internal jet flow at low flow rate for heightening the electric field intensity around the needle. We chose the PZT sol and silicone oil as the inner and outer flow, respectively. The jet stability generated by the nib will be higher than that caused by the ordinary nozzle and the jet’s length will be considered more prior to being broken. Coaxial EHD jet formation involves a variety of complex physical fields including electric field force, surface tension, viscous shear force, liquid internal pressure and gravity. The Multiphysics software (COMSOL, 5.2a, 2015, COMSOL, Stockholm, Sweden) was used to simulate and compare the flow velocity distribution and the voltage essential for stable jet formation when the auxiliary injection nib was placed. The needle added assisted-nib aims to enhance the electric field. It was scientifically examined via simulation analysis and experimental validation. Secondly, adding particle layer solution as the jet flow state inside the jet track indicator was used to observe the flow inside the liquid. For the first time, an experimental study observed that the lining of the coaxial jet solution inside space of coaxial electric jet produced the backflow phenomenon. Thus, the auxiliary metal nib can successfully reduce the backflow was proved. The uniform linear PZT nanowires and suspended nanobeam structures was successfully printed on silicon wafer and the substrate with channel. So, these printed structures can be used to provide a reference for the application of EHD technology in many M/NEMS sectors. This creates a new solution for smaller diameter jets to form micro- and nanostructures with optimizations printing diameters.

## 2. Printing Approach

Recent advances in fabrication technologies brought promising improvements in micro/nanoscale device applications. This allows the rapid printing of versatile low-cost micro- and nanostructures that were previously difficult or impossible to create and has enabled a wide range of applications such as flexible displays, optical sensors and high-performance devices. Despite recent improvements in printing techniques, electrohydrodynamic (EHD) printing can be optimized through theoretical analysis, simulations and experiments.

**Figure 1:**
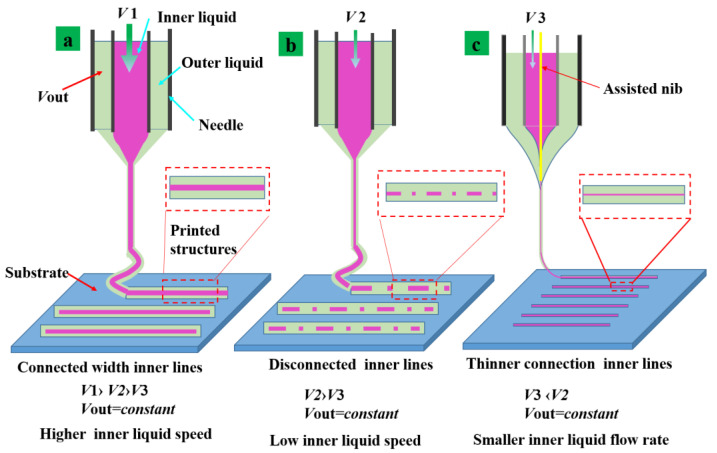
Printed structures shape change diagram. (**a**) Higher internal fluid flow rate v 1 (v_1_ > v_2_ > v_3_) while keeping out liquid and the applied voltage constant. (**b**) Lower internal fluid flow rate v 2(v_2_ > v_3_) (**c**) with assist nib and smaller inner liquid flow rate v3(v_3_ < v_2_).

A steady backstroke up inside the jet is the key to proper jet injection. Hayati, Barrero and Sheldon’s work on fluid motions inside a Taylor cone presented experimental results and theoretical modeling. There is a meridional recirculation motion during the EHD process as the fluid moves upward along the generator and starts it along the shaft. For the coaxial process, when the outer fluid drives the inner fluid in jet or is driven by the electric field force at the same time, the inner fluid will also flow back [35,42,43]. The fluid flow rate is the main parameter to control the size of the jet as shown in Figure 1a. However, the use of ultra-fine nozzles can easily cause clogging and cannot be used for material preparation. To overcome the above problems, this work discloses a novel nib-assisted coaxial EHD printing technique to reduce internal liquid reflux. Then, it increases the stability of the internal flow of the jet at low flow rates. A solid metal auxiliary nib was placed in the interior of a coaxial jet needle to improve the electric field intensity and the required voltage stability of the Taylor cone as shown in Figure 1. This is because the fluids inside and outside are driven by electric field injection at the same time. It is further driven by electric field injection through the role of fluid viscosity which brought about another type of fluid injection.

The jet shape can be interrupted in the space to make the fluid flow back to the surface of the internal parts and make the participation of the lining fluid to reduce the jet length as shown in Figure 1b. The added nibs extend the distance of the fluid acceleration which is accelerated by the force of the tangential electric field. The specific part of the potential difference was initially used to accelerate the jet for the deployment of the jet morphology. The potential difference can reduce the internal flow involved in the backflow at the same time and the smaller diameter size of the structures can be printed with a small flow rate as shown in Figure 1c.

## 3. Materials and Simulation Methods

### 3.1. Materials

**Figure 2:**
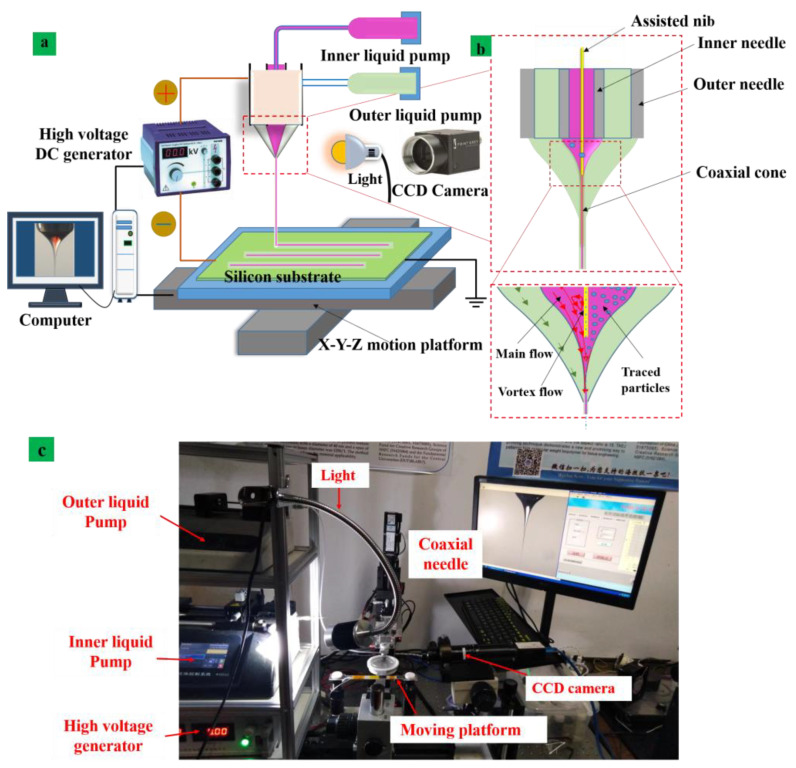
(**a**) The schematic diagram of coaxial electric jet equipment. (**b**) Local magnification of the assisted nib needle. (**c**) Photograph of device used for the printing experiment.

The schematic representation of the nib-assisted coaxial needle EHD device is shown in Figure 2a. The system contained an X-Y motion stage with high-motion precision, motion controller connected by a computer, a source of power supply and a CCD camera (Teledyne, Thousand Oaks, CA, USA). The high-voltage DC power supply generator (Tianjin Dong wen High Voltage Power Supply Plant, Tianjin, China) created a high electric field between the substrate and the coaxial needle. The force was caused by a high electric field that can eject functional inks from the nozzle [44,45]. The polyethylene particles with a diameter of 15 microns were selected to be the traced particles and the concentration was 5 percent. The study investigated that concentration that is too high will block the light and is, thus, challenging to observe, and concentration that is too low cannot accurately reflect the internal flow state of the fluid. The positive source of power supply was coupled to the coaxial nozzle and the negative pole was linked to the substrate (silicon). The silicon substrate was placed on controlled high-precision mobile platform and it was controlled by the motion controller with a computer (Lenovo, Beijing, China). A CCD camera was placed beside to witness the deposition process of lines and view the traced particles inside the coaxial cone. A local magnification of a coaxial injection needle with a fine metal nib inside the coaxial injection needle extending approximately from the end of the coaxial injection needle 100 µm is shown in Figure 2b. Moreover, Figure 2c represents the photograph of the device used for the experimental study.

### 3.2. Preparation of Materials and Substrate

In this study, the stainless steel coaxial nozzle consisted of an inner and an outer needle. The inner needle had an inner diameter of 130 µm and the outer width was 470 µm. Thus, the inner part of the outer needle was kept at 800 μm and the outer width was 1 mm. The assisted nib was tungsten wire and 50 μm in width, and the nozzle was held 1.8 mm away from the collector plate. The auxiliary metal needle was placed inside the coaxial spray needle. The silicone oil viscosity of the external solution was 60,000 CST; Because of its excellent piezoelectric properties [36], PZT sol is widely used as a solution for electropainting [41,46]. In this article, the 0.1 mol/L PZT sol was used as internal solvent material (prepared in our laboratory). The sol configuration process is illustrated in our previous work [47]. The properties of PZT sol such as surface tension were measured using a droplet shape analyzer (DSA100, Krüs GmbH, Hamburg, Germany) and viscosity was measured with a rotary viscometer (NDJ-79, Shanghai Pingxuan Scientific Instrument, Shanghai, China). The relative permittivity was obtained by precision instrument (4294A, Agilent Technologies, Santa Clara, CA, USA) and high viscosity silicone oil (viscosity: 60,000 cps, refractive index: 1.56) were selected as experimental materials in external fluids purchased from Merck (Merck Investment, Shanghai, China). The silicon wafers (Tianjin Semiconductor Technology Research Institute, Tianjin, China, resistance b 0.01) were selected as substrates and further ultrasonically cleaned with acetone to eliminate organic impurities. This was then cleaned with anhydrous ethanol and finally deionized (DI) water was used for sonication for 10 min. Then, it was baked at 120 °C for 30 min before the experiments. The characteristics of the materials used in this work are given in Table 1.

### 3.3. Characterization

The printed structures were characterized through an optical microscope (OLYMPUS STM6, Tokyo, Japan). A scanning electron microscope (SEM) (SU82200304070201, Hitachi High-tech, Tokyo, Japan) was used for characterization. A high-speed camera was used for characterization of printed structures (Factory production: pco.imaging, model: pco.dimaxS40316010502, Munich, Germany).

### 3.4. Simulation Model

**Figure 3:**
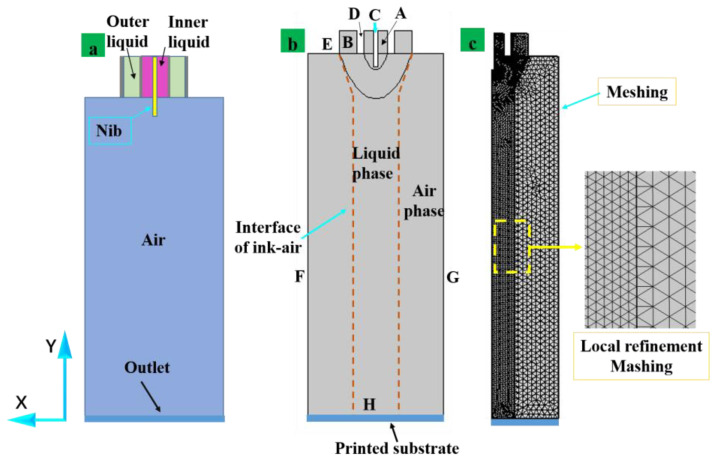
(**a**) Two-dimensional axisymmetric model structure, (**b**) phase field, (**c**) mesh diagram.

This paper adopted a two-dimensional axisymmetric model to solve a Multiphysics electrohydrodynamic problem; where A is the internal fluid in the internal nozzle (the material is PZT sol) and B is the external fluid in the nozzle (the material is silicone oil). Moreover, C is the auxiliary metal nib and D is the nozzle’s inner wall; E is the outer wall of the nozzle and F is the boundary. Figure 3a shows the two-dimensional pattern of nib-assisted coaxial EHD printing. The mesh was locally refined to reduce the amount of simulation computation and to ensure convergence of the computation in cells where the physical field in the phase field varied greatly, as shown in Figure 3c. To improve the convergence of the simulation, it is necessary to establish the initialization model. The two-dimensional axisymmetric model initialization condition patterns are shown in Figure 3b. The fundamental boundary conditions and the geometric model of the simulation are given in Table 2. This paper adopted a two-dimensional axisymmetric model to solve a Multiphysics electrohydrodynamic problem. The symmetry of the model and the result after meshing are shown in Figure 3c.

## 4. Results and Discussion

The finite element simulation analysis method was straightforward and efficient. It could further assist to determine the influence of various parameters on the formation of coaxial jet in the printing process. It could analyze the flow condition within the coaxial jet to provide guidance for the experiment. The cone jetting regime was investigated according to the geometric model, boundary settings and material properties. Based on the theory of the phase field method, the COMSOL Multiphysics setup was used to produce a field model of the electric coaxial jet. This study focused on the three critical points such as the formation process of the electric coaxial jet, the charge distribution and the flow condition inside the Taylor cone. The comparative analysis of the simulation and the experiments were then performed.

### 4.1. Velocity Field and Voltage Influence on the Coaxial Cone Jets Using Assisted-Nib and Normal Needle

**Figure 4:**
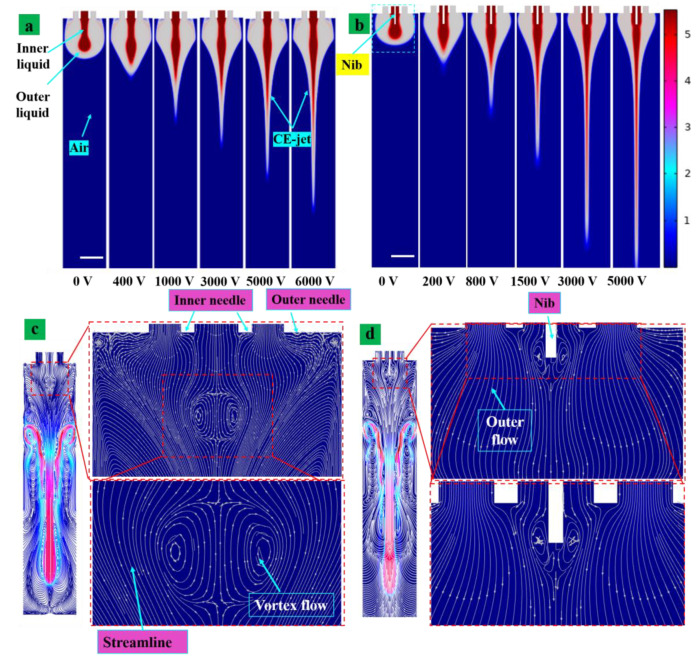
As the applied voltage increased, the coaxial jet morphology changes process diagram (**a**) common coaxial needle, (**b**) coaxial needle with an auxiliary nib, (**c**) flow field curve inside the coaxial Taylor cone without auxiliary needle, (**d**) internal liquid reflux reduce among the coaxial Taylor cone with auxiliary nib. Scale bar = 500 µm.

The coaxial cone jet flux formed by normal coaxial nozzle without using nib and assisted nozzle at different voltages is shown in Figure 4a,b. Figure 4a shows the simulation condition of the coaxial cone jet at different applied voltages of 0 kV, 400 V, 1 kV, 3 kV, 5 kV and 6 kV. The shape of the cone jet started to appear as the voltage increased. The simulation completed without adding a metal nib (Figure 4a) to achieve stabilized jet is the required voltage more than the auxiliary injection voltage, as shown in Figure 4b. The results indicate if there is no assisted-nib, the formation of jet was observed under voltage of 6 kV. So, adding the metal-assisted nib, the voltage required to form steady jet in the space was 5 kV. The flux was longer and the tip of the jet was thinner under the same voltage as the metal-assisted nib. By adding the extra nib, more fine-sized nanostructures print could be printed. This was due to the metal fiber coaxial metal nib end that increased the intensity of the local electric field and reduced the voltage required to form the stability of the Taylor cone. The fluid flowed across the surface of the auxiliary nib and was accelerated by the force of the tangential electric field. The stability of the jet generated by the end of an auxiliary metal needle was higher than that of the standard nozzle. The jet formation will have a considerable length before it breaks. In addition, the voltage was reduced, which avoids increasing the charge carried by the particles in the solution. The mutual repulsion between the charged particles and the evaporation of the solvent during the flight of charged particles in the jet was reduced. It further resulted in the decrease in the stability of a jet [24]. Using the internal fluid flow rate in the simulation was 150 nL/min and the external fluid flow rate was adjusted to 3 nL/min, respectively, at different time intervals such as 0.002 s, 0.004 s, 0.006 s, 0.008 s, 0.01 s, 0.012 s, 0.014 s and 0.016 s. Figure 4b illustrates the morphology of the jet with the auxiliary nib. The trends of the flow velocities field within the coaxial cone jet space are shown in Figure 4c,d. Within the coaxial cone jet, only a little internal fluid near the inner surface of the Taylor cone was accelerated to form the jet. Similarly, the fluid within the internal fluid was reversed. In addition, the external fluid filled the shape of the aircraft formation. Thus, the electric field caused the fluid at the interface to move along the tangential flow due to the interaction between the electric charges at the gas-liquid interface. Similar findings were reported by [31] through simulations from other studies. In the other study, there was no circulation in the fluid stream and the jet flow field was also unidirectional.

The reflux still existed in the inner solution of the coaxial Taylor cone as the auxiliary nib was added. However, the reflux was significantly reduced. The simulation results indicate that there was no reflux in the outer solution, regardless of the auxiliary nib and reflux only existing in the inner flow. The flow at the edge of the inner layer can be induced to participate in the jet flow due to the electric field shear force and viscous force of the outer liquid. The flow of the inner liquid part engages in the reflux recovery end along the midline symmetry axis and the edge part was divided into two parts after consumption. A unique part was entrained in the jet flow and the rest continued to reflux. The outer liquid (silicone oil) was accelerated to form a non-reflux jet instead of returning to the inside of the needle.

### 4.2. Space Charge Density of the Coaxial Cone Jets

**Figure 5:**
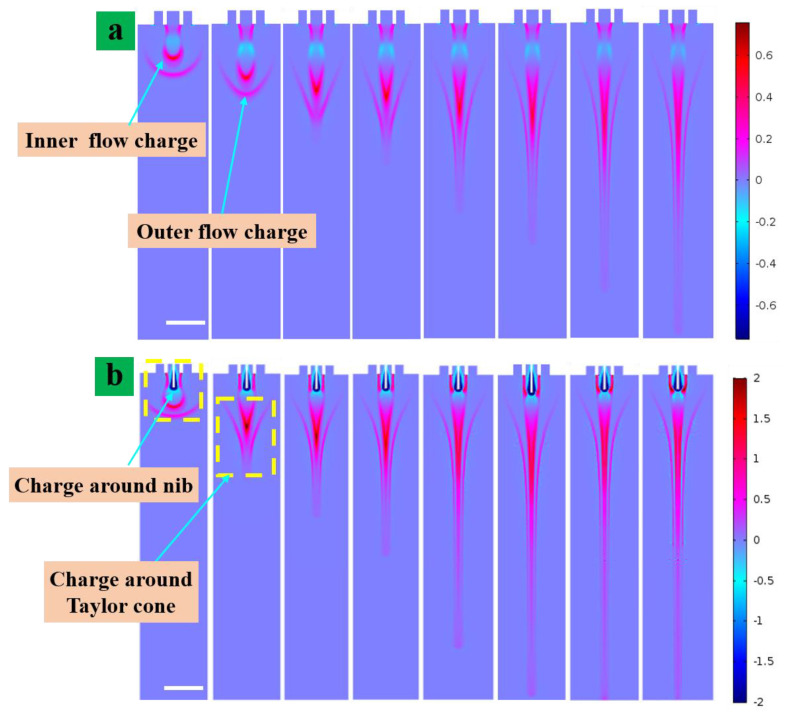
The space charge density of coaxial electric jet diagram at various times (**a**) common co −axial needle, (**b**) with auxiliary nib (1 ms, 3 ms, 5 ms, 8 ms, 11 ms, 14 ms, 17 ms and 20 ms). Scale bar = 500 µm.

The charge distribution is one of the essential aspects that affect the printing process and the size printed structures. Figure 5 shows the space charge density of the electric coaxial jet at different times. The scale bar in Figure 5 shows the influence of charge density. The maximum voltage value used in the simulation is illustrated in Figure 5a,b, and it illustrates the simulation of Taylor cone formation of two types of nozzles. Over time, the fluid evolved under the force of the electric field and a coaxial cone shape was formed. Figure 5a shows the simulation results of a normal coaxial nozzle which was without a metallic conductive nib inserted as an electrode. The jet gradually changed to form a meniscus through the rheology as a nozzle with a nib structure inside the nozzle under the action of the electric field force. Thus, the charge density on the meniscus gradually increased, so that the meniscus became a Taylor cone under the action of the electric field force. As the charge density increased further, the electric field strength also increased and the electric field overcame the surface tension of the liquid in the Taylor cone. The charge distribution matched the shape of the jet formation process very well according to the simulation results of the space charge distribution during the formation of the coaxial electric jet, as shown in Figure 5. The charge density of the external solution was mainly distributed on the surface of the external liquid. In contrast, the charge of the internal solution was distributed throughout the internal solution. The charge density in the inner solution was significantly higher than that of the outer liquid as the inner solution, especially before the voltage was applied to form a jet.

Furthermore, the charge density at the top of the Taylor cone reached a maximum. It will further decrease after the jet is formed. The pink area indicates high electric field strength. The charge was mainly visible in the inner fluid especially at the tip of the Taylor cone. The electric field stress was the dominant force and the acceleration of the jet was the largest. This was due to the versatile properties of the outer solution which was high viscosity non-conductive liquid silicone oil while the inner solution was PZT solution with good electrical conductivity. According to the internal thrust theory, the simulation results were also well verified in this work. The surface charge of the internal solution was greater and conduction occurred mainly through the internal solution. This is also consistent with the assumptions of other E-Jet printing works [30]. The highest charge density was achieved in the additional needle tip accessory and the charge properties were negative. It was similar to charge density while there was no current in the CFD model. In addition, the electric field strength at the end of the auxiliary nib was significantly higher than at other locations, indicating that the addition of the auxiliary metal needle increased the intensity of the local electric field. Adding an auxiliary metal nib to the system provides thrust to the auxiliary metal nib near the field region to further increase the electric field intensity of the system. Due to the same polarity of the same charge of the particles at the end of the injection, the needle formed the opposite polarity of the nearby charged particles. This working needle was injected into the anode where the auxiliary needle near the negatively charged particles is visible in the blue part as shown in Figure 5b. The charged particles in the jet were mainly positively charged particles of opposite polarity to the electrode which is represented in red. The distribution pattern of charged ions is consistent with that of the jet formation, which proves the correctness of the simulation results. Positively charged particles in the solution were repelled away from the spray needle and collect on the liquid surface under the action of surface tension.

## 5. Experiments of Nib-Assisted Enhancement Electric Field Intensity and Cone Jet Stability

**Figure 6:**
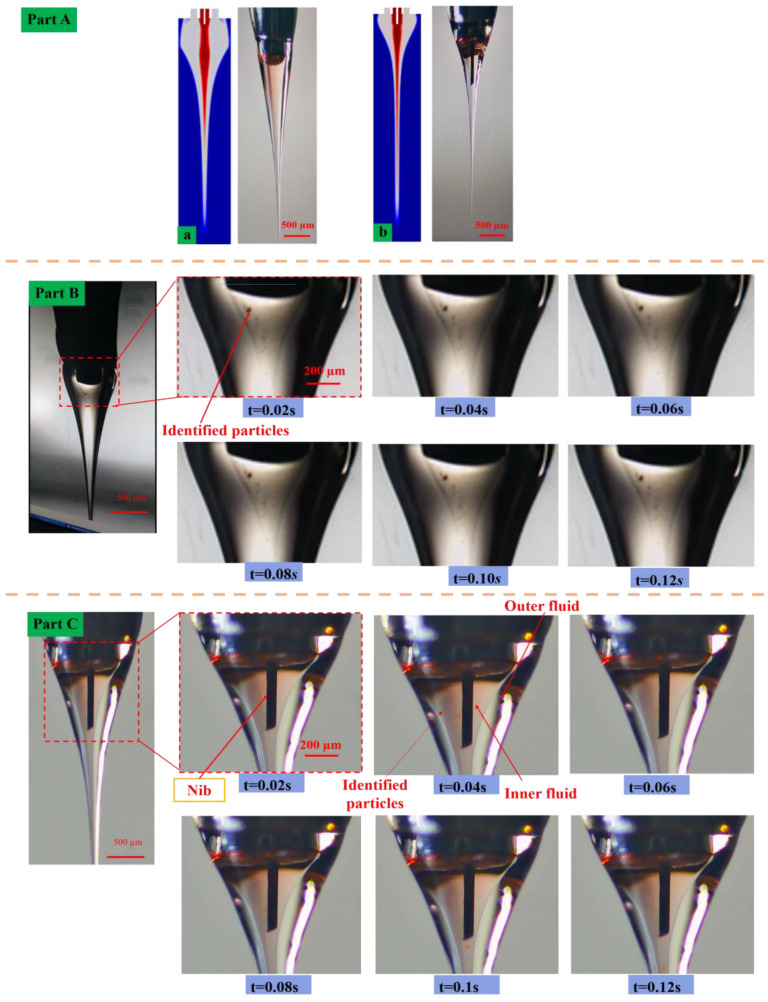
The identified particles inside the coaxial cone diagram at experiment variation at various times, (Part A) (**a**) traditional coaxial Taylor cone shape, (**b**) Taylor cone shape with an assisted nib, (Part B) the motion of polyamide12 particles in the cone images without nib assist as time change and (Part C) the motion of tracing particles in the cone images with nib assist at various time.

In this paper, we used polystyrene particles as tracking particles to verify the correctness of the simulation results and assumptions during the experiment. The internal solution of the coaxial electric jet is used as a transparent functional ink which is known as PZT material. The external solution is used as colorless silicone oil. To accurately reflect the state of fluid flow inside the Taylor cone, particle size was also the critical factor. If the particle size is too large, then it can be easy to block the needle. In addition, if the particle is too large, it will affect the flow regime between the tracer particles. Moreover, if it is too small, then it will not be easy to observe the phenomenon. Therefore, we preferred the polystyrene particle with a diameter of 15 µm as the tracking particle. The flow rate of the inner and outer layer solution used in the validation experiment was 200 nL/min and 3 µL/min, respectively. The silicone oil solution was chosen with a viscosity of 60,000 CST. The inner layer was PZT sol and the inner width of the inner injection needle was 200 µm. The outer diameter was 470 µm. The inner width of the outer needle was 800 µm and the height of the injection needle from the base was kept at 2.5 mm. The applied voltage was 6 kV, which follows the simulation parameters. To facilitate observation, a white lower Beijing plate was placed on the side of the jet as the light was beamed into the shape of the jet. The internal state of the jet was observed by the CCD camera, as shown in Figure 2c. The position of the tracking particles in the coaxial Taylor cone at different times without the auxiliary needle is shown in Figure 6a part (A). Similarly, the state of the particle flow in the coaxial Taylor cone with the auxiliary needle and the physical photograph of the experimental device are shown in part (A).

### 5.1. Experimentation of Particle Tracking Jet Reflux

The experimental results showed an annular vortex in the formation of the coaxial jet. The movement of polyamide particles in the coaxial cone with the time delay was 0.2 s between two consecutive images. Vortex formation and identified particles returning to the top of the inner fluid in the coaxial jet were visible for the first time. It was observed that the fluid flowing down the main path was separated by the annular vortex. At a bifurcation, most of the solution was withdrawn, returned to the central vortex, recirculated and mixed into the mainstream. The remaining liquid was drawn into the jet. The lycopene particles were chosen to study flow patterns in liquid cones and to photograph their dynamics [35]. An axisymmetric circulation pattern was observed within the Taylor cone. It was observed that the particles moved down the surface until they reach the tip of the Taylor cone, and then, they turned and moved up the line of symmetry. These findings are known as the phenomenon of backtracking in CFD results. The fluid passed through an imposed velocity to reach an even velocity a short distance downstream within the Taylor cone. This circulation was due to the action of Maxwell shear stress at the jet interface. It will draw a certain amount of fluid across the interface that cannot be depleted by the ejected thin jet.

The arrows indicate particles following the fluid flow within the jet as shown in Figure 6 (part B). However, the fluid inside the electric coaxial jet was different from that of the single injection needle, which is why the fluid outside the electric coaxial jet was all included in the jet without turning back. Parts in the internal solution that were in contact with the external solution were immiscible. The shear force in the electric field and the viscous shear force of the external fluid were responsible for forming the morphology of the jet. Once the edge part of the fluid was taken, it formed the pressure difference that made the fluid inside portion along the central axis of symmetry to form the circle. Likewise, after adding the auxiliary nib inside the needle surface which causes the liquid membrane to appear in a tangential force of the electric field, the extra potential jet liquid can be used to accelerate the flow. At the same time, the strong backflow blocks in smaller movements were reached at low flow or the ground below the threshold of injection voltage cone-jet oscillates. So, as a result of the existence of reflux with small flow rate in the liquid, the reduction in the length of the jet took place. Finally, it could not form stable jets because of the discontinuity in jet space. Therefore, we placed the conductive metal needle inside the coaxial injection needle as shown in Figure 6 (part C). The auxiliary metal nib cannot only reduce the participation in the reflux liquid volume but also increase the intensity of the local electric field. It reduced the tension needed to form a stable jet. It further accelerated the jet and the jet was too late to participate in the internal solution cycle. Then, it accelerated the jet spray formation and increased the acceleration distance.

Coaxial backflow exists in various places with single phenomena, but coaxial only exists in internal solution reflux. There was no reflow into the external solution. This is because the relaxation time of the electric field of the inner solution was much higher than the outer solution namely as the drive. The internal solution with the external solution of the fluid viscous force boundary directed the flow of the external solution. This force combined with the tangential force of the electric field accelerated the external solution of the formation jets. There was no backflow phenomenon in the outer solution. This not only reduced the difficulty of printing smaller sized structures but also avoided the problem of using a small diameter injection needle which was easy to become stuck inside the injection needle and challenging to process the injection needle. It increased the range of material use, reduced the difficulty of processing the injection needle and improved the printability of high-viscosity and low-flow solutions.

### 5.2. Printing Results and Characterization

#### Flexible Nanowires Deposition

**Figure 7:**
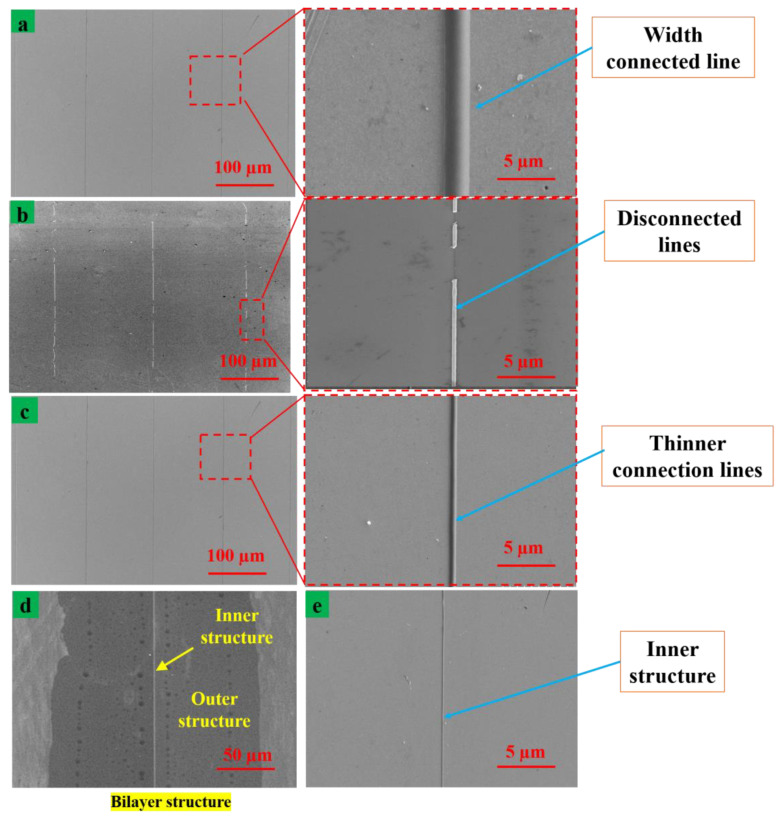
Experimental printing results of different flow rates of inner liquid and keep other process parameters unchanged. (**a**) Printed width structure with common coaxial needle at a large solution flow rate, (**b**) printed disconnected line structure without the auxiliary injection needle at a low solution flow rate, (**c**) printed thinner connected structure with the auxiliary nib injection needle at low solution flow rate, (**d**) a double-layer composite structure printed on a silicon substrate and (**e**) the remaining internal functional material after removing the outer silicone oil.

To verify the correctness of our hypothesis and simulation under the conditions of parameters such as voltage, the print speed and print height are kept constant. The printing experiment was compared with different internal flow rates (the internal solutions of 400 nL/min, 150 nL/min, and 50 nL/min, respectively); the flow rate of the external solution (3 µL/min) was kept constant. The experimental results of printing under different internal liquid flow rates using other constant process parameters are shown in Figure 7. The applied voltage was 5.5 kV, the substrate was moved at a speed of 300 mm/s.

The printed structures had good continuity but a large line width when the flow rate of the inner layer solution was 400 nL/min, as shown in Figure 7a. The width of the printed structure was about 400 nm after removing the outer silicone oil. The electron microscopy photograph of the detached structure was printed on the silicon substrate using an internal flow rate of 50 nL/min and the structures are shown in Figure 7b. Figure 7c illustrates the printing results when the inner layer volume and flow rate were adjusted to 50 nL/min together with the auxiliary metal nib injection needle. The flow rate decreased when the volume per unit time was constant. Thus, traffic reduction can reduce the size of printed structures with a flow rate of 50 nL/min. The size of the printed structures was reduced due to the internal resolution and the greater part of the resolution involved in the reversal. So, when the voltage was unchanged, the internal layer in the current of different solutions decreased and even existential interrupted the instability of the jet. The spray at the base of the structure represents a discrete discontinuous structure on the substrate. We placed the metal-assisted nib on the conductive injection needle, which not only greatly reduced the reflux flow but also increased the local electric field strength. Furthermore, it allowed more electrical tangential force to accelerate the jet and reduced the minimum current and voltage required for the stability of the cone jet.

Figure 7d shows the two-layer structure printed on the silicon substrate. It can be found that when the jet was pressed into the ground, the solution of the inner layer was completely solidified. The inner layer of the bilayer composite structure was formed by solidification of the inner layer solution in the center of the bilayer structure. Figure 7e shows the printed structure which was formed by the internal liquid. It was heated in isopropanol solution at 60 °C for 10 min to remove the external silicone oil and heated to 100 °C on the heating plate to make the solvent volatile. The remaining single layer structure had smooth surface and high straightness.

**Figure 8:**
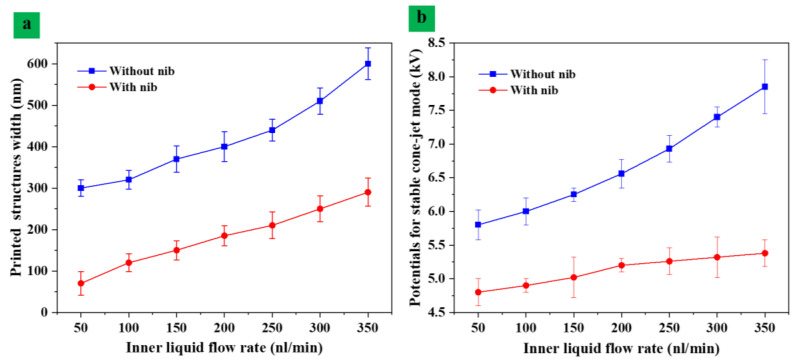
(**a**) Pattern width versus flow rate with and without assisted nib and (**b**) potential voltage for stable cone-jet mode.

Figure 8a shows the effects of the internal flow rate on the width of the print structure. The required potential voltage was used to maintain a stable cone-jet style was illustrate in Figure 8b. The regular nozzle and an assisted nib inside the nozzle were characterized in this work. The jet was accelerated by the tangential force of the electric field from the exit of the injection needle. The speed of the jet was most noticeable near the Taylor cone. When we reached the apex of the Taylor cone, the tangential electric field stress approached a maximum that was greater than the apex.. The auxiliary nib can increase the intensity of the local electric field and the tangential stress of the electric field. The more significant electric field stress accelerates the jet and further prolongs the acceleration time of the jet stream and reduces the diameter of the jet. Thus, it makes the printed structures on the substrate smaller in size. Figure 8a compares the diameter and size of the internal functional material from the needles of two different structures under different flow conditions. The diameter of the printed internal structures was mainly related to the flow rate. So, a series of experiments were conducted by setting the flow rate of the inner layer functional material to 50 nL/min, 100 nL/min, 150 nL/min, 200 nL/min, 250 nL/min, 300 nL/min and 350 nL/min, respectively. After removing the outer silicone oil, the printed structure consisted of inner liquid with diameters of 300 nm, 320 nm, 355 nm, 410 nm, 440 nm, 500 nm and 580 nm, respectively. In contrast, the size of the printed structure with the auxiliary nib was 80 nm, 110 nm, 145 nm, 170 nm, 210 nm, 230 nm and 260 nm, respectively. Consequently, the addition of the auxiliary needle can reduce the influence of the internal solution flow fluctuation on the stability of the jet and compare it with no auxiliary needle. The voltage required to form a steady stream was less than that of a traditional jet needle increasing the flow rate. The inner liquid flow rate was 50 nL/min, 100 nL/min, 150 nL/min, 200 nL/min, 250 nL/min, 300 nL/min and 350 nL/min and the voltage essential to formation of stable jet was 4.8 kV, 4.9 kV, 5.1 kV 5.35 kV, 5.5 kV and 5.8 kV, respectively, as shown Figure 8b. Similarly, without the use of a needle, the voltage of 6.2 kV, 6.5 kV, 6.7 kV and 6 kV, 6.5 kV, 6.9 kV, 7.2 kV and 7.5 kV were adjusted. Therefore, the voltage of the stable jet formation can be reduced by adding auxiliary needles. The jet flow stability can be increased by reducing the applied voltage using the needle plate.

**Figure 9:**
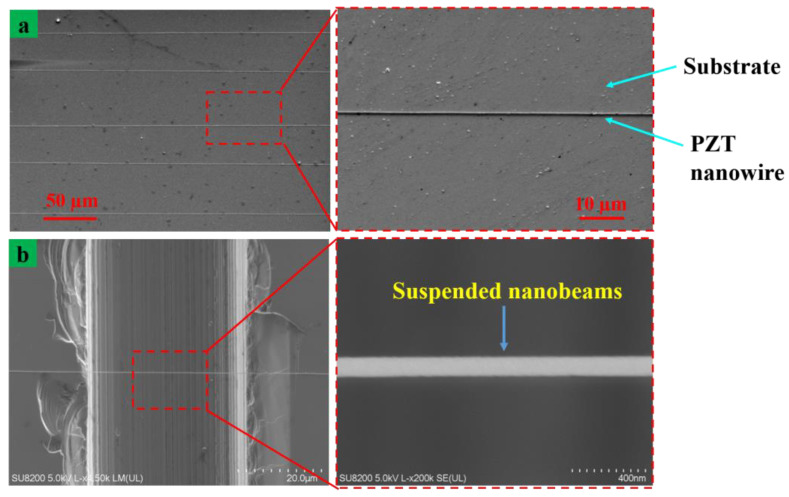
Printed structure using coaxial electric jet with auxiliary nib. (**a**) The array linear structure and (**b**) suspended beam structure on a pre-prepared substrate. Beside is a local magnification of the structure.

Figure 9 shows the printing structures on the silicon substrate with an internal liquid flow rate of 50 nL/min, voltage of 5.5 kV and substrate moving speed of 300 mm/s. The printing structure consisted of the inner functional material that remained after the outer silicone oil was removed, as shown in Figure 9a. On the right side of Figure 9a is the electron microscopic magnification of the local nanowire structure with a minimum size of about 70 nm. Figure 9b shows the structure of the simply suspended nanowire printed on the silicon wafer substrate with a pre-prepared channel. The channel was formed on the silicon wafer substrate with a diamond driver using a depth of about 60 µm and a width of 50 µm. Then, ultrasonic cleaning was carried out with deionized water and acetone for 10 min. Formerly, heating was carried out in the dryer at 120 °C for two hours for cleaning and drying. It can be seen in Figure 9b that the suspended beam structure had no suspended fracture phenomenon, a smooth surface and relatively uniform size was obtained. On the right side in Figure 9b is a local magnification of the hanging beam structure and its minimum diameter size can be up to 70 nm. The PZT material has piezoelectric properties along with extremely high aspect ratio and flexibility. It can be used to prepare sensitive piezoelectric sensors with nanobeams [39,40].

**Figure 10:**
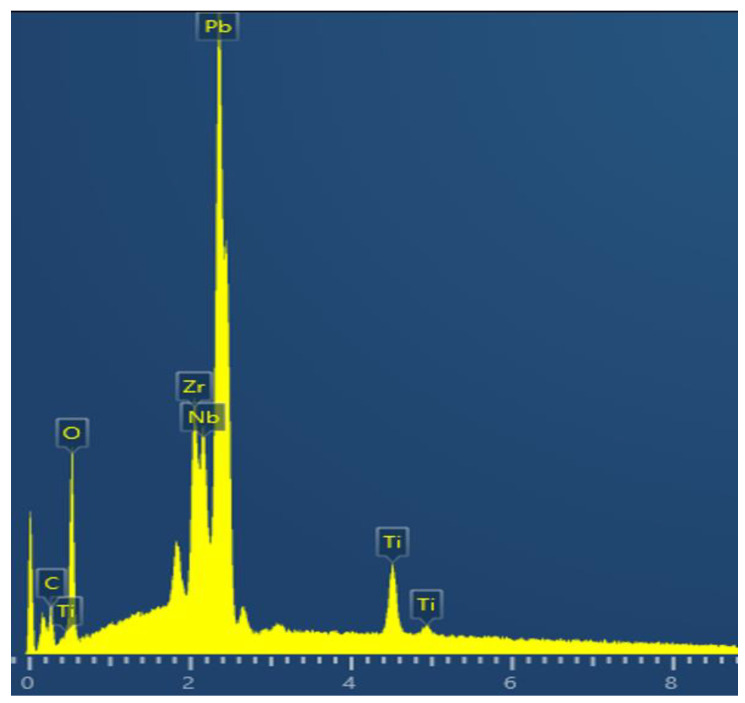
The EDS spectrum of the printed PZT nanostructures.

The printed PZT nanostructures were sintered in the muffle furnace at 650 °C for 20 min. Afterwards, the energy dispersive spectroscopy (EDS) measurement was executed and the results are shown in Figure 10. The chemical composition of the printed structure includes only “Pb”, “Zr”, “Ti” and “O” elements, which mean that the coating material was completely removed during the post-treatment process. We tested several samples and the results demonstrated an almost identical chemical composition of the elements.

## 6. Conclusions

In this paper, we designed a novel nib-assisted coaxial EHD printing technology to design and print nanowires. It was associated with the conventional EHD printing process. Therefore, it defeated the complex processing of using thinner nozzles to form smaller patterns with a low tracking rate and the delinquent instability when using low-viscosity printing fluid. For the first time, reflux in the internal solution of the coaxial electrojet was experimentally observed and simulated. Furthermore, the effect of nib-assisted electric field strength enrichment was studied by simulation. It was found from the simulation that by adding an assisted nib, a smaller steady-state voltage and thin jet morphology can be obtained. The simulation and experimental results were analyzed to observe the change in fluid flow inside the jet. In this work, we used particle tracking of the coaxial jet backflow to investigate the visual missing interior solution of the search. The experiment revealed that coaxial jets exist in internal solution reflux and the internal solution reflux speed is measured by a series of experiments. An auxiliary metal nib was placed inside the coaxial interior and then successfully reduced the internal fluid backflow and realized the small flow rate from the stable coaxial jets. The stable flux was formed under low voltage conditions and the size of the printing structure was reduced. It further provided a reference for realizing regular printing with a small solution flow for M/NEMS application.

## Figures and Tables

**Table 1:** The liquid properties used for coaxial electrohydrodynamic printing.

Properties Values	Inner (PZT Sol)	Outer (Silicone Oil)
Viscosity	160 mPa·s	58.56 Pa·s
Specific gravity	1016 kg m^−3^	976 kg m^−3^
Surface tension	0.0193 N/m	0.022 N/m
Dielectric constant	22	2.7

**Table 2:** Frontiers of the flow field and the electric field in the model.

Boundary	Electrostatic Field	Hydrodynamic Field
A: Inner needle inlet	E = V_0_	U_i_ = Q_i_/A_i_
B: Outer needle inlet	E = V_0_	U_o_ = Q_o_/A_o_
C: Wall of auxiliary needle	E = V_0_	U = 0
D: Wall of inner needle	E = V_0_	U = 0
E: Wall of outer needle	E = V_0_	U = 0
F: Boundary of computational domain	E = V	U = 0
G: Boundary of computational domain	E = V_0_	P = 0
H: Outlet of needle system	0	P = 0

## Data Availability

Not applicable.
